# Higher Sensory Sensitivity is Linked to Greater Expansion Amongst Functional Connectivity Gradients

**DOI:** 10.1007/s10803-022-05772-z

**Published:** 2022-10-13

**Authors:** Magdalena del Río, Chris Racey, Zhiting Ren, Jiang Qiu, Hao-Ting Wang, Jamie Ward

**Affiliations:** 1 School of Psychology, University of Sussex, Brighton, UK; 2 Sackler Centre for Consciousness Science, University of Sussex, Brighton, UK; 3 Sussex Neuroscience, University of Sussex, Brighton, UK; 4 School of Psychology, Southwest University, Chongqing, China; 5 Laboratory for Brain Simulation and Exploration (SIMEXP), Montreal Geriatrics Institute (CRIUGM), University of Montreal, Montreal, Canada

**Keywords:** Autism spectrum disorders, Sensory sensitivity, fMRI, Functional connectivity

## Abstract

Insofar as the autistic-like phenotype presents in the general population, it consists of partially dissociable traits, such as social and sensory issues. Here, we investigate individual differences in cortical organisation related to autistic-like traits. Connectome gradient decomposition based on resting state fMRI data reliably reveals a principal gradient spanning from unimodal to transmodal regions, reflecting the transition from perception to abstract cognition. In our non-clinical sample, this gradient's expansion, indicating less integration between visual and default mode networks, correlates with subjective sensory sensitivity (measured using the Glasgow Sensory Questionnaire, GSQ), but not other autistic-like traits (measured using the Autism Spectrum Quotient, AQ). This novel brain-based correlate of the GSQ demonstrates sensory issues can be disentangled from the wider autistic-like phenotype.

**Supplementary Information:**

The online version contains supplementary material available at 10.1007/s10803-022-05772-z.

The understanding and diagnostic definition of the neurodevelopmental condition of autism spectrum disorder (ASD) has evolved considerably during the last decades (e.g., Verhoeff, 2013). A parsimonious account of the complex and heterogeneous disorder remains elusive, with different theories highlighting various aspects of the phenotype, such as atypical sensory processing (Happé & Frith, 2006; Mottron et al., 2006), social and communication deficits (Baron-Cohen et al., 1985), or atypical inference (e.g., Brock, 2012; Palmer et al., 2017; Pellicano & Burr, 2012; Sinha et al., 2014; Van de Cruys et al., 2013).

The spectrum perspective on ASD has led to the wide-spread study of autistic-like traits in the general population. A substantial proportion of these studies quantifies the degree of autistic-like traits by means of one summary index, commonly the total score of the Autism Spectrum Quotient (AQ; Baron-Cohen et al., 2001). This measure, which quantifies various characteristic traits of ASD, is continuously distributed in the general population (Constantino & Todd, 2003). However, further traits have been associated with the autistic(-like) phenotype which are not captured by the AQ, notably sensory atypicalities. It is important to distinguish between different levels of such sensory processing differences, and note that the scale used here, the Glasgow Sensory Questionnaire (GSQ; Robertson & Simmons, 2013) measures sensory issues primarily at the level of subjective sensory sensitivity (Ward, 2019) or affective reactivity (He et al., 2022), as opposed to, e.g., objective or perceptual sensitivity, and we therefore refer to this level of analysis throughout in using the term sensory sensitivity. Whilst the nomenclature used in the sensory processing literature is largely inconsistent, with researchers using the terms ‘sensitivity’, ‘reactivity’, and ‘responsivity’ interchangeably, there is no evidence of a strong association between these different levels (e.g., Schulz & Stevenson, 2022) or a clear model of their interaction, making the distinction between these constructs instrumental to furthering our understanding of variability in sensory processing. Sensory issues are more commonly experienced by individuals with ASD yet are also present to a lesser degree among the neurotypical population and are not necessarily associated with a diagnostic condition (Horder et al., 2014; Little et al., 2017; Robertson & Simmons, 2013; Van Hulle et al., 2012), whereby there is evidence of sensory issues having a non-linear relationship with the AQ (e.g., Sapey-Triomphe et al., 2018), in line with the documented enhanced impact of sensory issues on the daily lives of individuals with ASD, which can be debilitating (e.g., Robertson & Simmons, 2015). Sensory issues at the level of affective reactivity are also prominent in other neurodevelopmental and/or psychiatric groups. For example, sensory sensitivity is associated with a history of mental illness, trait anxiety and migraine (Horder et al., 2014) as well as ADHD (Bijlenga et al., 2017), Tourette syndrome (Belluscio et al., 2011), and synaesthesia (Ward et al. 2017a). While the GSQ was designed to target the specific type of sensory issues associated with ASD, it correlates highly with alternative sensory processing scales, such as the Adolescent/Adult Sensory Profile (Brown et al., 2001), which was not developed with the aim of capturing issues most characteristic of ASD (Pearson’s r = 0.64 in Horder et al. (2014)). Together, this suggests that a symptom-level, diagnosis-agnostic approach is viable, and overall, parsing the autistic(-like) phenotype into different dimensions allows for a more fine-grained and mechanistic perspective of autism. In fact, applying this approach to resting state functional magnetic resonance imaging (rs-fMRI) data has proven fruitful in previous clinical studies, e.g., focusing on social vs non-social processing (e.g., Di Martino et al., 2009), restricted and repetitive behaviours (e.g., Bertelsen et al., 2021), and sensory issues (e.g., Green et al., 2016; Keehn et al., 2021).

Here we evaluate individual differences in connectivity derived from non-clinical rs-fMRI data related to autistic-like trait variability with an emphasis on sensory sensitivity. Each participant’s functional correlation matrix describes the undirected functional connectivity of each cortical region with every other region. As such, each row of the matrix contains a vector representing the functional correlation of a given cortical region to every other region. Gradient decomposition analysis generates lower-dimensional representations of connectivity (‘cortical gradients’) based on the pattern of covariance in the matrix. It groups cortical regions by identifying the major axis of variation of all regions’ connectivity vectors using Principal Component Analysis (PCA), and then projecting each region’s vector onto this major axis (Margulies et al., 2016). Each region’s gradient score is thus defined as its distance along this major axis of variation in connectivity. In other words, regions with similar connectivity profiles (similar vectors) have similar gradient values and are closer together along the gradient. The principal gradient (i.e., the gradient that explains most variance) typically describes a continuum from sensory cortex to transmodal default mode networks, dovetailing with classic primate tract-tracing findings (Mesulam, 2012). Although the default mode network has been more commonly described as mediating perceptual decoupling or disengagement from the external world (Raichle et al., 2001), mind-wandering (Christoff et al., 2009), and self-referential processing (Gusnard et al., 2001), recent accounts drawing on a growing body of work have linked the network to abstract cognition more broadly (Smallwood et al., 2021). In this framework, it is thought that the principal gradient reflects a functional hierarchy which enables the flexible integration and segregation of sensory signal processing and more abstract, higher-order cognitive functions (Murphy et al., 2018).

Cortical regions are assigned a priori to one of seven atlas-defined cortical functional networks (Schaefer et al., 2018), i.e., independently of the gradient decomposition. Each network is thus composed of a subset of cortical regions with corresponding gradient scores. These collectively define a distribution of gradient scores per network whose number of data points is equal to the number of regions in the network. Various scalar parameters can be used to characterise these networks' gradient distributions in terms of their central tendencies, or to characterise the sum of all networks' gradient distributions in terms of its variance. These parameters can then be used to compare networks.

A previous gradient mapping study in clinical ASD has shown a compressed principal gradient, such that unimodal and transmodal regions are less functionally distant in ASD than in neurotypical controls (Hong et al., 2019). Complementary stepwise functional connectivity (SFC) analyses in the same study revealed accelerated connectivity transitions from sensory to association cortices, followed by unsuccessful transitions to transmodal regions, such that the transmodal core is dissociated from the periphery. Overall, this atypical macroscale organisation might explain symptoms in ASD ranging from difficulties with simple sensory stimuli, such as photosensitivity, to higher-order cognition deficits, as are socio-communication problems.

Taking a trait-based approach, we therefore asked, first, if the functional hierarchy compression found in clinical cases of autism extends to individuals with autistic-like traits in the general population, as measured using the AQ. Second, we ask if differences in macroscale functional connectivity are related to the dimension of sensory sensitivity. Specifically, we investigate functional integration/segregation by means of various metrics that capture distance (i.e., similarity in functional connectivity profiles) along the connectivity gradients, while focusing on the default and visual networks (the ones typically found to be furthest apart). Evaluating the dimension of sensory sensitivity independently of other autistic-like traits is particularly relevant to the interpretation of the atypical connectivity in ASD outlined above—variation in functional connectivity related to non-clinical sensory sensitivity may shed light on the variation related to the more complex autistic(-like) phenotype, as these sensory issues are not necessarily accompanied by additional higher-order symptoms in the general population.

Briefly, we do not find evidence of the clinical findings in Hong et al. (2019) extending to a neurotypical sample, as gradient compression was not associated with the AQ. Nonetheless, we find that sensory sensitivity is associated with an expansion of the connectome hierarchy and particularly with the differentiation of functional connectivity patterns of the default and visual networks. Our results thus point to variability across various dimensions of autistic-like traits in a neurotypical sample. It remains to be tested if and how these findings will manifest in clinical autism, or, alternatively, if there is a categorical difference between individuals with high levels of autistic-like traits and clinical autism. Further, this strongly suggests the need for a more nuanced understanding of the behavioural correlates of integration/segregation patterns found in clinical as well as non-clinical populations.

## Methods

### Participants

401 participants (111 male, aged 18–26 years, mean = 21.04 years, standard deviation = 1.27) participated in this research as part of a project investigating associations among genes, brain imaging and mental health (Liu et al., 2017). Data collection was in accordance with the Declaration of Helsinki (Association 1991). All participants were right-handed, with no history of neurological or psychiatric problems. All participants provided written informed consent and received payment for their time. The study was approved by Southwest University (Chongqing, China) Brain Imaging Centre Institutional Review Board.

31 participants were excluded due to missing data or poor structural or functional data quality (23 had poor structural contrast leading to poor surface reconstruction, 5 had missing or corrupt data, 3 generated incomplete correlation matrices). The final sample for the analysis therefore comprised 370 participants (99 male, aged 18 to 26 years, mean = 21.05, standard deviation = 1.29).

**Fig. 1:**
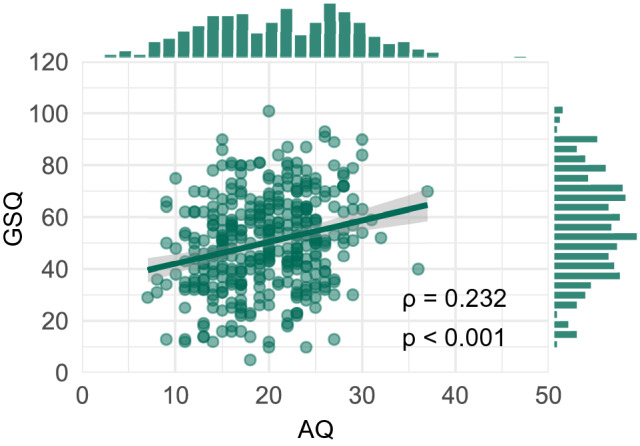
Distribution of the main behavioural measures of interest (total AQ and GSQ scores) and their correlation, in our final sample of 370 participants

Despite this being an opportunistic non-clinical sample of primarily female college students, the AQ and GSQ distributions include a broad range of scores (M_AQ_ = 19.6, SD_AQ_ = 5.3; M_GSQ_ = 50.1, SD_GSQ_ = 18.2; see Fig. 1). For comparison, the mean AQ score in a typical sample drawn from a non-clinical population was estimated to be approximately 17, and the pooled standard deviation to be 5.6 (Ruzich et al., 2015), lower than the mean AQ in our sample. Mean GSQ scores likewise tend to be higher in clinical autism than in non-clinical samples (e.g., M_GSQ_ = 75.8, SD_GSQ_ = 24.9 in Ward et al. (2017b)), yet there is nonetheless some overlap.

### Behavioural Measures

Participants completed the Chinese versions of the Autism Spectrum Quotient (AQ; Baron-Cohen et al., 2001; translation in Lau et al. (2013)) and the Glasgow Sensory Questionnaire (GSQ; Robertson & Simmons, 2013; translation in Ward et al. (2021)).

The questionnaire data for the full sample of 401 participants was analysed in detail in Ward et al. (2021), where the reliability of the AQ, Cronbach’s alpha, was reported to be 0.633. The AQ consists of a total of 50 items belonging to 5 subscales (“Social skill”, “Communication”, “Attention switching”, “Imagination”, and “Attention to detail”). Responses are given on a 4-point Likert scale (“Definitely Agree”, “Slightly Agree”, “Slightly Disagree” and “Definitely Disagree”), yet these are commonly subsequently binarized, such that final scores range from 0 to 50 (though see e.g., Austin (2005) for a continuous scoring method).

In the same full sample, the reliability of the GSQ, Cronbach’s alpha, was 0.907 (Ward et al., 2021). The GSQ consists of 42 items, with six items pertaining to each of seven sensory modalities (vision, hearing, olfaction, taste, touch, proprioception and vestibular sense) and targeting hyper- and hyposensitivity equally. Responses are given on a 5-point scale (“Never” = 0, “Rarely” = 1, “Sometimes” = 2, “Often” = 3, “Always” = 4), resulting in a range of final scores from 0 to 168.

### MRI Data Acquisition

Rs-MRI data were acquired at the Brain Imaging Centre of Southwest University, China, using a 3.0-T Siemens Trio MRI scanner with a 16-channel whole-brain coil (Siemens Medical, Erlangen, Germany). For each subject, 242 functional images were acquired with a gradient echo type echo planar imaging (EPI) sequence [echo time (TE) = 30 ms; repetition time (TR) = 2000 ms; flip angle = 90 degrees; slice thickness = 3.0 mm; slices = 32; resolution matrix = 64 × 64; voxel size = 3 × 3 × 3 mm]. Participants were instructed to lie down, close their eyes without falling asleep, and rest whilst not thinking about any particular thing and keeping their head still for the duration of the 8-min scan.

### Preprocessing

Preprocessing was performed using a pipeline combining tools from the SPM12 suite (Wellcome Department of Imaging Neuroscience, London, UK), FSL (Smith et al., 2004) and Freesurfer (Fischl, 2012).

#### Anatomical Preprocessing

T1-weighted anatomical volumes were corrected for gradient nonlinearities based on scanner calibration measurements. Non-brain data were removed from each T1 volume using the FMRIB Brain Extraction Tool (Jenkinson et al., 2005; Smith, 2002), then aligned to the Montreal Neurological Institute (MNI) 152 standard template anatomical image using FSL’s FMRIB's Linear Image Registration Tool with 12 degrees of freedom (Jenkinson & Smith, 2001; Jenkinson et al., 2002). Each volume was inspected for image artifacts and tissue contrast and rejected if deemed of poor quality. Cortical surface representations were generated from the T1 volume (1-mm resolution) using FreeSurfer version 6 beta (build-stamp 20161007) with the *-hires* option. Transfer mappings to fsaverage space were calculated and various standard atlases were mapped for each subject. Final surface visualizations were consolidated into movies, to be visually inspected. Some subjects showed poor Freesurfer surface generation, with poor segmentation leading to voxels from skull, CSF, or cerebellum being assigned to cortical surface reconstructions. This is due to distortions or poor contrast in the raw structural data and would result in added noise when mapping functional data to cortical grey matter. 23 subjects with poor surface reconstructions were excluded from further analysis.

#### Functional Preprocessing

Both temporal and spatial preprocessing was performed on the fMRI data. First, cubic interpolation was performed on each voxel’s time-series data to correct for differences in slice acquisition times and to obtain an integer sampling rate of 1.0 s (acquisition TR = 2 s). Rigid-body motion parameters were estimated from the undistorted EPI volumes using the SPM12 utility *spm_realign*. Finally, cubic interpolation was performed on each slicetime-corrected volume to compensate for the combined effects of EPI distortion and motion. The first 10 volumes of each EPI run were discarded. No spatial smoothing or temporal filtering was performed. The average of the preprocessed functional volumes was coregistered to the T1 anatomical volume (affine transformation estimated using a 3D ellipsoid that focuses the cost metric on cortical tissue). This resulted in a transformation that maps the EPI data to the participant-native brain anatomy volume. Functional data were then re-processed in cortical surface space. Identical procedures associated with volume-based preprocessing were performed, except that the final spatial interpolation was performed at the locations of the cortical surface vertices. The use of simple interpolation to map volumetric data onto surface representations helps maximize spatial resolution and avoids making strong assumptions about cortical topology. All resting state gradient analyses were performed on pre-processed data projected into fsaverage space for each subject.

### Gradient Derivation

Each participant’s motion traces obtained during preprocessing were regressed out of the time-series in fsaverage space. The time-series were then parcellated using the Schaefer et al. (2018) atlas with 400 parcels assigned to 7 global networks and averaged per ROI. Pearson correlation matrices were generated per subject, z-transformed, averaged across subjects and the average correlation matrix z-to-r-transformed.

Gradients were extracted using the Brainspace Toolbox (Vos de Wael et al., 2020). We calculated the first ten group-level gradients for our sample, using PCA as the dimension reduction technique, a sparsity parameter of 0.95 and a cosine similarity kernel. Linear dimension reduction methods, such as PCA and more conservatively thresholded functional connectivity matrices, have been found to produce more reliable gradients, which would in turn predict behavioural data more accurately (Hong et al., 2020). Although here we focus on the first gradient, it has been shown that extracting ten gradients increases the degree of fit between the group-averaged gradients and the individual-level gradients, quantified as the mean r-to-z-transformed Spearman rank correlation between individual and average gradients (Mckeown et al., 2020). We subsequently calculated the first ten individual-level gradients using identical parameters while Procrustes-aligning them to the group-average gradients. This alignment step brings the connectivity pattern into a common space, allowing for comparability within the current sample.

In order to evaluate the generalisability of the dimensionality reduction results, we additionally calculated the first ten group-level gradients for a subsample of the HCP dataset freely available within the Brainspace toolbox (n = 217, 122 female, mean age = 28.5, standard deviation = 3.7; for further details see Vos de Wael et al., 2018). The correspondence between gradients was assessed through Fisher’s r-to-z-transformed Spearman’s rank correlation. We calculated the correlation between each of the first three individual-level gradients and the group-level gradients, between the first three group-level gradients and the HCP-derived group-level gradients, and between each of the first three individual-level gradients and the HCP-derived group-level gradients (see Supplementary Tables 1–3).

### Individual Differences in Connectivity Related to Autistic-like Traits and Sensory Sensitivity

For visualisation purposes, the principal gradient scores for each participant were divided into 50 bins, and the average count per bin across participants plotted for the entire sample as well as for subsamples based on questionnaire scores. The same procedure was conducted after segregating the principal gradient scores according to the Schaefer atlas 7-network functional parcellation (Schaefer et al., 2018). Note that all statistical tests were conducted using the full sample of 370 participants and the gradient scores for the 400 parcellated regions from the Schaefer atlas, not the histogram bins.

In Hong et al. (2019), one of the main features differentiating the connectome in ASD from that in controls was the degree of integration/segregation. The unsupervised derivation of connectivity gradients was validated in their study with graph theory-derived methods. The distance between the two ends of a given connectivity gradient conceptually represents network integration/segregation. Using stepwise functional connectivity analysis, they quantified this distance using the number of steps between the two poles of the principal gradient (including both direct and indirect connections). Other ways of measuring gradient compression and network segregation/integration focusing on different network properties of functional connectomes have been proposed in the recent literature. We show that our own pattern of results is not critically dependent on the choice of measurement: conceptually related metrics yield largely consistent results. Metrics of network segregation/integration applied in the current study and their implications are listed below.

1. Network median distance: The difference between the median gradient score value of two networks. This measure captures the distance between two given networks along the principal gradient (in one-dimensional space, i.e., independently of any of the remaining gradients), independently of the relative position of any other networks.
2. Gradient range and variation: the difference between the maximum and minimum gradient score, and the standard deviation of the gradient scores, respectively (Xia et al., 2020). This measure is also restricted to the principal gradient, and is both more global and more coarse-grained, because (1) it does not differentiate between networks, and (2) it is less susceptible to differences in the shape of the underlying gradient score distribution (e.g., the skew would not affect these metrics as it would the mean of the networks). This limits assumptions and would also be advantageous if the relative position of networks is expected to vary.
3. Network peak distance (Bethlehem et al., 2020): The difference between the peak gradient scores of two networks. This metric differs from the network median distance only in the method to characterise the gradient distribution, as it relies on the findpeaks function in Matlab’s Signal Processing Toolbox instead of the median. This metric might be less appropriate if the underlying distributions are relatively flat, as the peak may not be estimated optimally. On the other hand, the peak may be a preferable measure of central tendency if the distribution is extremely skewed.
4. Eccentricity (Valk et al., 2021): the distance between each region and the centre of the gradient coordinate system spanned by gradients 1 through 3 per participant (i.e., in three-dimensional space). The average value per network is a measure of its functional integration/segregation, where high values indicate segregation and lower values integration. More specifically, it captures how distinct the patterns of connectivity of a given network are on average relative to the overall connectome hierarchy (a schematic illustration can be found in Fig. 6). This metric would thus be more appropriate if one is interested in the integration/segregation of a single given network within the overall hierarchy. An additional factor to take into account is that this metric is calculated in three-dimensional space. By incorporating two additional gradients, it may be more comprehensive, in that the distances here capture connectivity similarity on three axes of variation.
5. Dispersion (Bethlehem et al., 2020; Valk et al., 2021): Within-network dispersion is defined as the sum squared Euclidean distance of each region’s gradient score from the network centroid (the median of the network gradient values). It thus captures the similarity of the functional connectivity profiles within a given network. As in the case of eccentricity, it may be a more appropriate metric if one is interested in a single given network, independently to its relation to the overall hierarchy or any other networks. Between-network dispersion is defined as the Euclidean distance between network centroids and is thus equivalent to the network median distance in three-dimensional space. (Schematic illustrations can be found in Fig. 6). It therefore provides a measure of the differentiation of two given networks from one another. The same considerations regarding one- vs three-dimensional space would apply for both dispersion metrics as for eccentricity.

Our hypothesis was related specifically to the networks with the highest distance from each other, the visual and the default mode network. As part of an exploratory analysis, we also calculated the distance between every other network combination and global measures, where applicable. The metrics listed above were investigated in relation to individual differences in autistic-like traits. Spearman’s rank correlation was calculated between each metric and the AQ and GSQ score. Given the tight relationship between the computed metrics, we also report Spearman’s rank correlation between our novel connectivity distance metric and the previously proposed metrics. As a control analysis, we additionally tested the correlation between the AQ and GSQ scores and the variance explained by the principal gradient.

### Characterisation of Subject Head Motion

In order to characterise effects of subject motion during functional scans, two summary measures of motion were computed in line with the recommendations of Power et al. (2012, 2014). The first was Framewise Displacement (FD). This is calculated as the median of the timeseries of absolute values of the derivatives of the twelve realignment parameters computed during preprocessing. The mean of these twelve values per participant is used as a summary measure of the change in head position throughout the functional scan. A second was DVARS (DV), which was computed volume-to-volume as the root mean squared of the derivatives of the time-courses within a whole brain mask of the functional timeseries data. This measure is designed to summarize high amplitude BOLD signal changes that co-occur with head movement events. FD and DV measures of motion were computed after initial data preprocessing, and prior to functional connectivity processing.

The median FD values range between 0.005 and 0.072 mm, i.e., all participants’ average degree of motion was below the 0.2 mm cut-off used in previous studies (Parkes et al., 2018; Power et al., 2013). It is possible that individual differences in autistic-like traits, including sensory sensitivity, result in variation in motion within the scanner (e.g., due to the unfamiliar and loud environment of the scanner). We thus examined the relationship between summary measures of motion and both gradient metrics and behavioural measures of sensory sensitivity. We did not find any significant correlations of the head motion metrics with total AQ (FD: ρ_s_ =  − 0.029, p = 0.581; DVARS: ρ_s_ = 0.060; p = 0.253) or total GSQ (FD: ρ_s_ =  − 0.082, p = 0.114; DVARS: ρ_s_ = 0.041, p = 0.427). Perhaps unexpectedly, if anything the general trend is of negative correlations of FD and sensory sensitivities, regardless of whether hyper- and hyposensitivities are evaluated separately. We nonetheless report the results of linear models including the measures of head motion as regressors together with the pre-planned correlations.

Analysis scripts and pre-processed data are available on GitHub: https://github.com/magdadelrio/SensoryGradients.

## Results

### Gradient Derivation

We applied an unsupervised non-linear dimensionality reduction technique to functional connectomes derived from rs-fMRI in a sample from the general population (Fig. 1A). This decomposes the data into gradients, where the principal gradient corresponds to the component in the connectivity data which explains the most variance. A given region’s distance from another within a particular gradient (i.e., the difference in score) reflects the degree of similarity in connectivity patterns between the two regions. This is visualized with the colour map, such that regions with more similar connectivity profiles are depicted with similar colours and regions with more dissimilar connectivity profiles are depicted with more dissimilar colours. It should be noted that the positive and negative scores are arbitrary, as this measure is not directed.

**Fig. 2:**
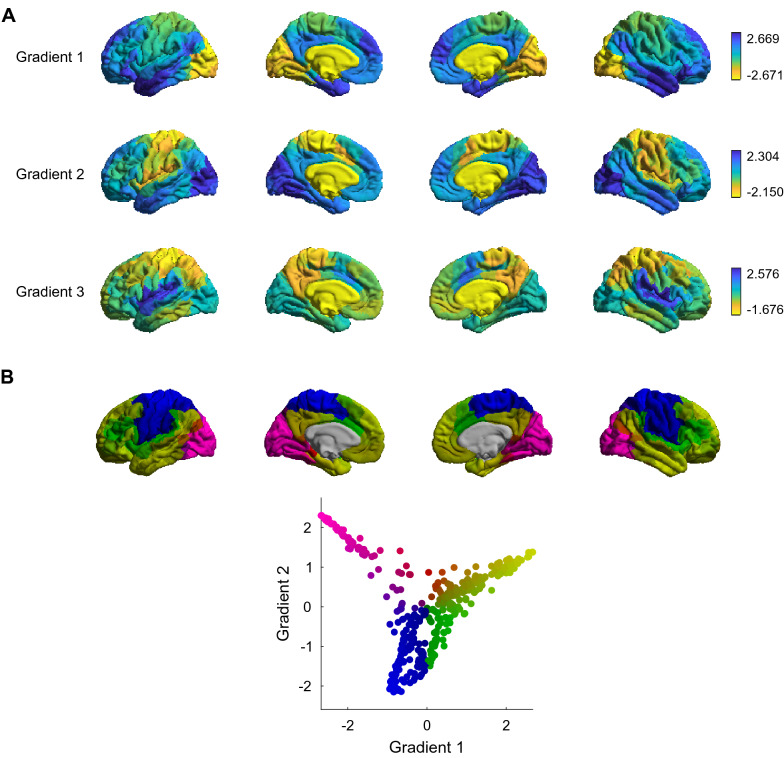
**A** First three gradients derived from our sample’s group-average functional connectivity matrix, using PCA as the dimension reduction technique, a sparsity parameter of 0.95 and a cosine similarity kernel. Regions with similar connectivity patterns are in close proximity along the gradient and thus share similar gradient scores, represented here as colours. **B** First two gradients derived from our sample’s group-average functional connectivity matrix visualized concurrently in Euclidean space as well as on the cortical surface

Unimodal regions (e.g., visual cortex) and transmodal regions (e.g., angular gyrus, midfrontal gyrus, inferior frontal gyrus) constitute opposite ends of the principal gradient. The second gradient spans from somatomotor to visual areas, and the third gradient, from somatomotor to ventral attention areas. Figure 2B shows the first two gradients (i.e., the two components that explain the most variance in the connectivity data) plotted concurrently in Euclidean space. As in Fig. 2A, regions with more similar connectivity patterns are depicted with similar colours, whereby the additional dimension provides more nuanced information regarding the similarity of two given regions’ connectivity profiles, as it can be assessed relative to two axes of variation.

We were primarily interested in the first gradient, previously associated with differences between ASD and typically developing individuals (Hong et al., 2019), yet note that the second gradient accounts for a similar level of variance, as in other datasets (for the scaled eigenvalues of each component, or gradient, for our sample’s group-averaged low-dimensional connectivity representation, see Supplementary Fig. 2E).

### Gradient Characterisation

**Fig. 3:**
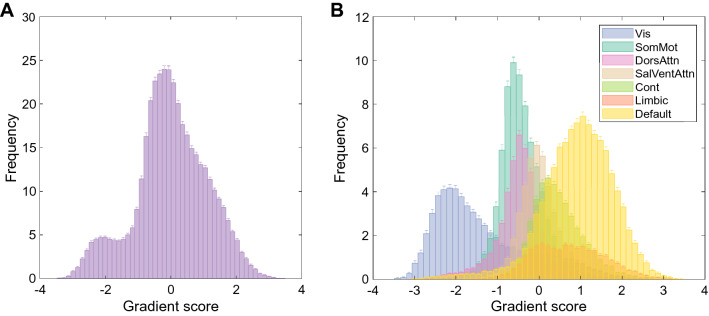
**A** Global histogram of the principal gradient scores for the full sample. Regions with similar connectivity patterns are in close proximity along the gradient and thus have similar gradient scores. **B** Histogram of the principal gradient scores for the full sample segregated into the 7 networks identified by the Schaefer atlas (*Vis* visual, *SomMot* somatomotor, *DorsAttn* dorsal attention, *SalVentAttn* salience-ventral attention, *Cont* control, *Limbic* limbic, *Default* default mode network). Error bars represent the standard error of the mean bin count

The distribution of the principal gradient scores for the entire sample can be seen in Fig. 3A, with the gradients segregated by network in Fig. 3B. This shows that the bimodality of the distribution in our sample is primarily driven by the segregation of the visual network from the remaining networks, whereby the default and limbic networks are most distant from it.

**Fig. 4:**
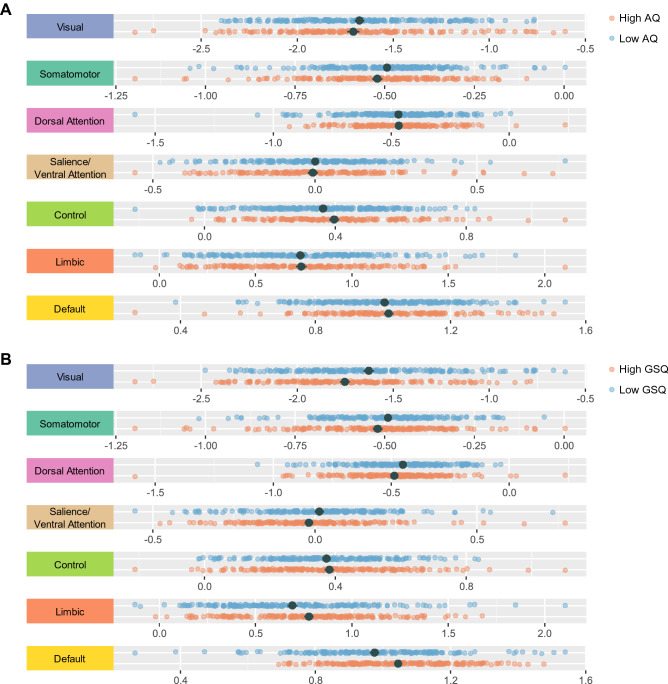
**A** Distribution of the median gradient scores segregated by the 7 networks of the Schaefer atlas for participants with high (AQ > 20, n = 161), and low AQ scores (AQ ≤ 20, n = 209) as per a median split. **B** Distribution of the of the median gradient scores segregated by the 7 networks of the Schaefer atlas for participants with high (GSQ > 51, n = 185), and low GSQ scores (GSQ ≤ 51, n = 185) as per a median split. Regions with similar connectivity patterns are in close proximity along the gradient and thus have similar gradient scores. Mean and standard error are depicted in dark grey

While Hong et al. (2019) show an overall compression of the principal gradient in clinical ASD, we do not find similar evidence of this being the case in individuals with high autistic-like traits (see Fig. 4A). The central peak appears minimally more right-skewed for high vs low AQ participants, which can be traced back primarily to the default network by segregating the distribution by network (see Supplementary Fig. 2A). Conversely, variability in GSQ scores is reflected in shifts in most of the networks (see Fig. 4B and Supplementary Fig. 2B).

To quantitatively assess the individual differences in connectivity related to autistic-like traits, we computed several metrics characterising the gradient distribution and network integration/segregation, based on the clinical findings in Hong et al. (2019) (see Methods section *Individual differences in connectivity related to autistic-like traits and sensory sensitivity*).

#### Network Median Distance

**Fig. 5:**
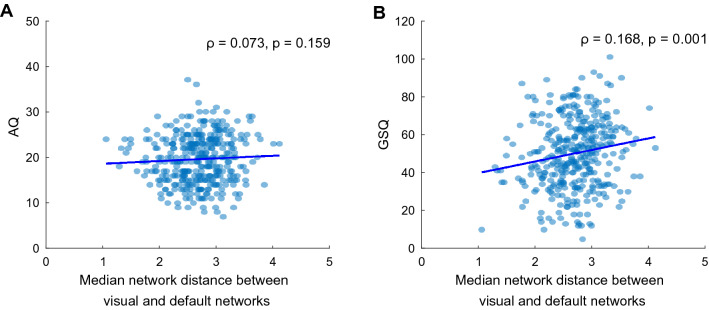
Correlation between the distance from the median of the default mode network gradient score to the median of the visual network gradient score and **A** total AQ score, and **B** total GSQ score. Each dot represents an individual participant. The continuous line shows a least-squares linear fit of the data

The network median distance between the visual and the default mode network served as the primary proxy for the degree of compression of the principal gradient. Counter to our prediction, there was no significant correlation between this metric and the total AQ score (ρ_s_ = 0.073, p = 0.159, Fig. 5A). However, we do find a significant correlation between this metric and the total GSQ score (ρ_s_ = 0.168, p = 0.001, Fig. 5B). It is still significantly predicted by GSQ after accounting for age, gender and head motion indexed as FD and DVARS in a linear model, though it should be noted that FD is also a significant predictor in the opposite direction (β_AQ_ = 0.002, p = 0.647; β_GSQ_ = 0.004, p = 0.007; β_FD_ = − 13.09, p = 0.008; β_DVARS_ = 2.984 × 10^−4^, p = 0.833). Follow-up correlations show that the effect seems to be driven by an expansion of the gradient at both poles: the median gradient score of the DMN increases with sensory sensitivity (ρ_s_ = 0.151, p = 0.004) and the median gradient score of the visual network decreases with sensory sensitivity (ρ_s_ = − 0.123, p = 0.018). Including age, gender and head motion in a linear model does not change the pattern of results for the median of the visual network (β_AQ_ = − 0.001, p = 0.890; β_GSQ_ = − 0.003, p = 0.025; β_FD_ = 3.506, p = 0.376; β_DVARS_ = 4.745 × 10^−5^, p = 0.967), or the default network, although FD again has a significant and opposite effect in this case (β_AQ_ = 0.002, p = 0.352; β_GSQ_ = 0.001, p = 0.013; β_FD_ = − 9.586, p < 0.001; β_DVARS_ = 3.459 × 10^−4^, p = 0.510).

As an exploratory analysis, we also investigated the relationship between autistic-like traits and the pairwise distance along the principal gradient between all other network combinations. Once again, we find no significant correlations with AQ scores (see Table 1). On the other hand, with higher sensory sensitivity, the default network is increasingly distant (i.e., more segregated) from all other networks but the limbic network, and the limbic network is increasingly distant from all other networks but the control network (see Table 2).

**Table 1:** Spearman’s rank correlation between AQ scores and distance between the medians of the principal gradient by network

	Visual	Somatomotor	Dorsal attention	Salience/ventral attention	Control	Limbic
Somatomotor	0.018					
Dorsal attention	0.008	− 0.028				
Salience/ventral attention	0.03	− 0.014	0.026			
Control	0.067	0.08	0.073	0.1		
Limbic	0.005	− 0.008	− 0.002	− 0.013	− 0.035	
Default	0.073	0.094	0.082	0.073	0.019	0.062

*p < 0.05, **p < 0.01, ***p < 0.001

**Table 2:** Spearman’s rank correlation between GSQ scores and distance between the medians of the principal gradient by network

	Visual	Somatomotor	Dorsal attention	Salience/ventral attention	Control	Limbic
Somatomotor	0.077					
Dorsal attention	0.072	0.008				
Salience/ventral attention	0.081	− 0.02	0.04			
Control	0.099	0.056	0.07	0.087		
Limbic	0.131*	0.124*	0.140**	0.120*	0.1	
Default	0.168**	0.153**	0.150*	0.155**	0.107*	− 0.041

*p < 0.05, **p < 0.01, ***p < 0.001

#### Gradient Range and Variance

The GSQ shows significant correlations with the principal gradient range (ρ_s_ = 0.121, p = 0.020) and the gradient variation (ρ_s_ = 0. 179, p < 0.001). The same is not found for the AQ (gradient range: ρ_s_ = 0.050, p = 0.342; gradient standard deviation: ρ_s_ = 0.071, p = 0.173). The finding concerning the global variance holds after accounting for age, gender and head motion in a linear model, with head motion indexed as FD as a significant predictor in the opposite direction (β_AQ_ = 0.001, p = 0.658; β_GSQ_ = 0.001, p = 0.010; β_FD_ = − 3.401, p = 0.024; β_DVARS_ = 5.230 × 10^−4^, p = 0.218), yet the same cannot be said for the global range, where GSQ is no longer a significant predictor alongside AQ, while FD is (β_AQ_ = 0.004, p = 0.505; β_GSQ_ = 0.002, p = 0.169; β_FD_ = − 13.592, p = 0.024; β_DVARS_ = 0.002, p = 0.238). As the default and visual networks are the maximally distant networks in connectivity space in our sample, the median distance between these networks is itself strongly correlated with the overall gradient range (ρ_s_ = 0.580, p < 0.001) and overall gradient standard deviation (ρ_s_ = 0. 729, p < 0.001).

#### Network Peak Distance

The relationships between the peak distance (Bethlehem et al., 2020) between the visual and default networks and the AQ and GSQ scores are not significant (AQ: ρ_s_ = 0. 052, p = 0.322; GSQ: ρ_s_ = 0. 031, p = 0.553), the linear model showing the same result (β_AQ_ = 0.001, p = 0.730; β_GSQ_ = 2 × 10^−4^, p = 0.704; β_FD_ = 1.893, p = 0.455; β_DVARS_ = 0.001, p = 0.218). However, we find a low correspondence between the peak and the median, particularly in the default network (default network: ρ_s_ = − 0. 090, p = 0.073; visual network: ρ_s_ = 0. 290, p < 0.001), and therefore also a lower correspondence between the two distance metrics (ρ_s_ = 0. 357, p < 0.001). While this should perhaps temper any conclusions from the results, one could argue that the median is more robust to differences in distribution shape than the peak.

#### Eccentricity

**Fig. 6:**
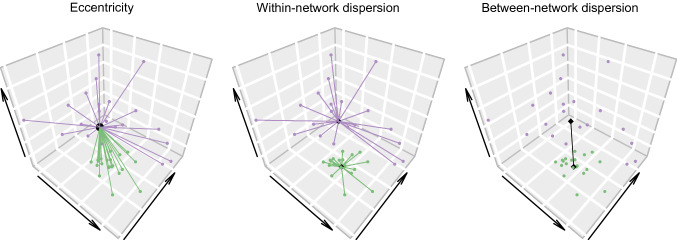
Proposed metrics of functional integration/segregation address different aspects of connectivity within and across networks. Filled green and purple circles represent the vertices (cortical regions) that respectively form each of two mock (brain) networks, black rhomboids represent the network centroid (the median gradient score of a given network for the first three gradients), and the large black circle represents the origin of the coordinate system. The lines indicate the distances computed in each metric: within-network dispersion is the squared sum of Euclidean distances of each vertex from the corresponding network centroid (the variability in gradient scores, i.e., connectivity profiles, of a given network—how differentiated are the connectivity profiles within a given network); between-network dispersion is the Euclidean distance between network centroids (the average similarity of the gradient scores, i.e., connectivity profiles for two given networks—how differentiated are the connectivity profiles of two given networks from one another); eccentricity is the squared sum of Euclidean distances of each vertex from the origin (average similarity of the gradient scores, i.e., connectivity profiles, of a given network from the overall hierarchy—how differentiated are the connectivity profiles relative to the overall hierarchy)

In line with the distance metric we propose (network median distance), individuals who score higher on the GSQ have more segregated default networks on the eccentricity measure (Valk et al., 2021, illustrated in Figure 6). The control and limbic networks, which fall on the same end of the principal gradient as the default network, are also significantly more segregated in individuals with higher sensory sensitivity, as is the gradient taken as a whole (see Table 3), whereby the linear model confirms this pattern for global eccentricity (β_AQ_ = 0.001, p = 0.424; β_GSQ_ = 0.001, p = 0.034; β_FD_ = − 1.275, p = 0.345; β_DVARS_ = 1.226 × 10^−4^, p = 0.345) and the eccentricity of the limbic network (β_AQ_ = − 0.001, p = 0.738; β_GSQ_ = 0.002, p = 0.016; β_FD_ = − 1.906, p = 0.460; β_DVARS_ = 0.002, p = 0.024), but not for the eccentricity of the default network (β_AQ_ = 0.003, p = 0.122; β_GSQ_ = 0.001, p = 0.118; β_FD_ = − 5.705, p = 0.001; β_DVARS_ = 0.001, p = 0.224). None of the networks’ eccentricity correlate significantly with the AQ.

**Table 3:** Spearman’s rank correlation between AQ and GSQ scores and mean eccentricity, averaged by network and across all networks (global) – the seven networks are presented in rank order along the principal gradient

	AQ	GSQ
Visual	0.052	0.095
Somatomotor	− 0.01	0.073
Dorsal attention	0.004	0.012
Salience/ventral attention	0.071	0.067
Control	0.039	0.107*
Limbic	0.05	0.145**
Default	0.088	0.148**
*Global*	*0.069*	0.155**

*p < 0.05, **p < 0.01, ***p < 0.001

Values in italics relate to global measures

#### Dispersion

Within-network dispersion (Bethlehem et al., 2020, illustrated in Fig. 6) does not correlate significantly with AQ scores (see Table 4). The limbic network has higher within-network dispersion in individuals with higher GSQ scores (see Table 4), however this is not robust when accounting for motion (β_AQ_ = 0.089, p = 0.604; β_GSQ_ = 0.066, p = 0.183; β_FD_ = − 199.530, p = 0.253; β_DVARS_ = − 0.125, p = 0.012). There are also no significant correlations between AQ and between-network dispersion (Bethlehem et al., 2020, illustrated in Fig. 6) for any network combination (see Table 5). Conversely, individuals with high GSQ show increased segregation of the default network from the somatomotor and visual networks, as well as of the limbic network from the dorsal attention and visual networks (see Table 6). Focusing on the networks of interest, the linear model shows the same pattern for the visual and default networks (β_AQ_ = 0.002, p = 0.672; β_GSQ_ = 0.004, p = 0.019; β_FD_ = − 9.297, p = 0.080; β_DVARS_ = −4.030 × 10^−4^, p = 0.790) as well as the limbic and visual networks (β_AQ_ = −0.004, p = 0.580; β_GSQ_ = 0.004, p = 0.023; β_FD_ = 2.399, p = 0.710; β_DVARS_ = 8.468 × 10^−4^, p = 0.790). In other words, these network pairs are not only more functionally distant along the unimodal-to-transmodal axis, but also in the three-dimensional space spanned by the first three gradients. In fact, the network median distance and the between-network dispersion for the visual and default network correlate strongly (ρ_s_ = 0.977, p < 0.001).

**Table 4:** Spearman’s rank correlation between AQ and GSQ scores and within-network dispersion, the seven networks are presented in rank order along the principal gradient

	AQ	GSQ
Visual	0.068	0.090
Somatomotor	− 0.065	0.018
Dorsal attention	− 0.015	− 0.050
Salience/ventral attention	0.024	0.010
Control	0.007	0.078
Limbic	0.086	0.106*
Default	0.045	0.047

*p < 0.05, **p < 0.01, ***p < 0.001

**Table 5:** Spearman’s rank correlation between AQ scores and between-network dispersion, calculated pairwise for all seven networks

	Visual	Somatomotor	Dorsal attention	Salience/ventral attention	Control	Limbic
Somatomotor	− 0.014					
Dorsal attention	− 0.01	0.06				
Salience/ventral attention	0.038	− 0.062	0.035			
Control	0.046	0.083	0.069	− 0.006		
Limbic	0.002	0.014	0.003	0.046	− 0.018	
Default	0.061	0.091	0.053	0.035	0.032	0.008

*p < 0.05, **p < 0.01, ***p < 0.001

**Table 6:** Spearman’s rank correlation between GSQ scores and between-network dispersion, calculated pairwise for all seven networks

	Visual	Somatomotor	Dorsal attention	Salience/ventral attention	Control	Limbic
Somatomotor	0.055					
Dorsal attention	0.056	− 0.015				
Salience/ventral attention	0.057	− 0.051	− 0.009			
Control	0.072	0.088	0.064	0.021		
Limbic	0.116*	0.091	0.145**	0.073	0.093	
Default	0.137**	0.119*	0.149	0.064	0.076	0.006

^*^p < 0.05, **p < 0.01, ***p < 0.001

One may wonder whether these differences emerge because data from individuals with higher sensory sensitivity are harder to fit to the principal gradient, yet the variance explained by the principal gradient does not correlate positively with either AQ (ρ_s_ = 0.099, p = 0.056) or GSQ (ρ_s_ = 0.095, p = 0.067), with the linear model supporting these results (β_AQ_ = 0.057, p = 0.275; β_GSQ_ = 0.014, p = 0.343; β_FD_ =  − 99.527, p = 0.061; β_DVARS_ =  − 0.011, p = 0.481). Although this is not a significant relationship, the overall organization of the connectome along the principal gradient is, if anything, a better fit in individuals with high levels of these traits. The results are strong enough to survive a Bonferroni correction for 42 tests (21 network pairs × 2 behavioural measures, namely AQ and GSQ), ρ_s_ = 0.168, p = 0.001 being below the Bonferroni-adjusted threshold of 0.0012. We would note that this is a very conservative correction in the present case given that the behavioural measures, network pairs and neuroimaging metrics, which are putatively measuring a similar latent construct of integration/segregation, are not independent.

In summary, increases in sensory sensitivity are linked to greater dispersion of the principal gradient of the functional connectome. This arises in particular due to a greater segregation (less integration) between visual and default mode networks, the endpoints of the principal gradient. The finding is robust across nearly all measures of dispersion; namely, network median distance between visual and default networks, overall SD of the principal gradient, and measures of eccentricity and dispersion, whereby motion might be a confound for the global range of the principal gradient and the eccentricity of the default network. The only conceptually similar measure to not show this pattern was network peak distance (between visual and default networks), which fails to account for the shape of the distributions.

## Discussion

In the current study we use a dimensionality reduction method to relate individual differences in macroscale whole-brain resting connectivity organisation (cortical gradients) to differences in traits related to autism in the general population. These traits are assessed by two self-report measures: the AQ, a summary measure of general autistic-like traits, and the GSQ, an index of sensory sensitivity. While we do not find evidence of differences in the compression of the principal gradient associated with the AQ in our non-clinical sample, we do find evidence of variability associated with the specific dimension of sensory sensitivity. Specifically, higher sensory sensitivity is correlated with an expanded functional connectome hierarchy, such that unimodal and transmodal regions are more segregated from each other.

Focusing on sensory sensitivity has the advantage of reducing the dimensionality of traits of interest linked to the autistic-like phenotype. Here we find individual differences that are robust against the specific choice of functional integration/segregation metric: the default network and visual network are more segregated from one another in individuals with higher sensory sensitivity. The more coarse-grained measures (eccentricity, range and variation of the principal gradient) show increased overall segregation in high sensory sensitivity, whereby range appears to be confounded by head motion. Assessing segregation at the network level indicates that it is nevertheless not a global effect, as not all network pairs show increased segregation. Rather, increased segregation is most prominent for the default and visual networks, though other network pairs, such as the limbic and visual networks, are also significantly more segregated. In addition, within-network dispersion results suggests that whereas specific networks are more segregated from each other, the individual regions within the networks are not. The main findings are similar in three-dimensional space, as evidenced by the metrics of eccentricity and between-network dispersion, implying the increased segregation is not neutralized by additional axes of variation. Specifically, this holds for the increased segregation of the visual and default networks, the visual and limbic networks, the somatomotor and default networks, and the dorsal attention and limbic networks. Increased segregation of the somatomotor and limbic networks, dorsal attention and default networks, salience/ventral attention and default networks, and salience/ventral attention and limbic networks are only apparent in the principal gradient, i.e., connectivity patterns are only significantly more dissimilar with sensory sensitivity along the first extracted dimension. Among all the employed measures of integration between visual and defaults networks, the network peak distance is the only one that does not confirm the correlation. This is however not surprising, given the limitations of the peak in the characterisation of wider-spread distributions, such as the default or limbic network distributions.

In sum, the expansion of the gradient can be traced back primarily to the increased distance of the visual network from default and limbic networks. Abnormal activity in the limbic network, which supports emotion processing and memory, has been previously linked to sensory over-responsivity (e.g., Green et al., 2016). This network overlaps with the default mode network, which has been linked to numerous ‘higher’ cognitive functions, including semantic processing (Binder et al., 2009), association formation (Bar et al., 2007), self-referential processing (Gusnard et al., 2001), self-generated thought (Benedek et al., 2016), episodic memory (Rugg & Vilberg, 2013), autobiographical planning (Spreng et al., 2015), and social cognition (Iacoboni et al., 2004). More generally, it has been proposed to support abstracted and generalizable representations which integrate features from unimodal regions (Murphy et al., 2018). More distinct connectivity patterns may thus reflect lower integration of bottom-up and top-down signals in individuals with higher sensory sensitivity (for an analogous interpretation of gradient contraction, see Xia et al. (2020)). We speculate that the reduced integration is related to the processing of bottom-up inputs in the absence of an integrated context, i.e., with the lack of prediction, generalization, and abstraction leading to the known difficulties filtering out certain sensory signals.

As the GSQ assesses both hyper- and hyposensitivities for seven sensory modalities, it is reasonable to suppose that this scale indexes a highly multidimensional construct. One may additionally question if the principal gradient, which involves exclusively visual cortex, as opposed to all unimodal cortices, can be clearly interpretable in relation to the GSQ as a multimodal measure. However, factor analyses tend to show the GSQ measures a singular latent factor (Robertson & Simmons, 2013) or, at most, two factors corresponding to hyper- and hyposensitivity (in the French version of the GSQ: Sapey-Triomphe et al., 2018). Indeed, hyper- and hyposensitivities in various sensory modalities tend to co-occur within the same individuals (our sample reports both hypersensitivities, with subscale scores ranging from 4 to 53, M_Hyper_ = 28.86; SD_Hyper_ = 9.86, and hyposensitivities, with subscale scores ranging from 0 to 48, M_Hypo_ = 21.25; SD_Hypo_ = 9.46; the correlation between hyper- and hyposensitivity subscales being ρ_s_ = 0.765, p < 0.001; and between vision and all other sensory modalities, ρ_s_ = 0.744, p < 0.001). Our previously published data using the GSQ on a clinical autism sample showed a large effect size for visual hypersensitivity (Cohen’s d = 0.83), which is similar to that for hypersensitivity for audition (Cohen’s d = 1.14), gustation (Cohen’s d = 0.82), olfaction (Cohen’s d = 0.71), and touch (Cohen’s d = 1.09; Ward et al., 2017b). This does not imply all sensory modalities are equally common (indeed the effect sizes are larger for audition and touch, as expected from the autism literature), yet, together with the findings from factor analyses, the pattern in the data does suggest that there is a strong association between the senses and an underlying common factor tapped into by the GSQ.

In line with the behavioural data structure, our principal measure of integration/segregation (default and visual network median distance) correlates with both hyper- and hyposensitivity, whereby the effect size is slightly higher for the hyposensitivity subscale (hypersensitivity: ρ_s_ = 0.136, p = 0.009; hyposensitivity: ρ_s_ = 0.181, p < 0.001). Although we would be reluctant to draw any strong conclusions from this difference, given the tight association between the subscales, further work could address how functional connectivity variability maps onto different aspects of sensory sensitivity. For example, it is noteworthy that the GSQ items targeting hyposensitivity often involve sensory-seeking behaviour or self-stimulation (e.g., ‘Do you flick your fingers in front of your eyes?’), such that it may be important for future work to distinguish between affective reactivity and behavioural responsivity, which are currently conflated in the majority of sensory scales, and potentially include measures of restricted and repetitive behaviours alongside measures of sensory issues. Regarding the distinction between sensory modalities, we can further show that our principal finding using default and visual network median distance holds if we remove the vision-related items from the GSQ score (ρ_s_ = 0.157, p = 0.003), i.e., it is not critically dependent on atypical visual sensitivity. In this context it is also noteworthy that the principal gradient consistently ranges from the default mode network to the visual cortex, and not any other primary sensory cortices—in other words, the distance metric examined here was determined by the results of the dimensionality reduction technique. Overall, there are multiple ways in which the GSQ scores can be broken down for exploratory analyses, yet this is outside the scope of the current paper—the relevant data and statistical analysis code is available on GitHub, allowing for it to inform future work on specific testable hypotheses, as opposed to the data-driven methods used here, for instance, by investigating stepwise functional connectivity seeded from other primary sensory cortices.

We do not find evidence that the atypical functional hierarchy in Hong et al. (2019) extends to individuals with high autistic-like traits as measured by the AQ. Our null finding regarding the AQ should be interpreted with caution, given (1) that any differences in connectivity driven by individual differences in subclinical autistic-like traits would be more subtle than those in clinical populations, and could thus require substantially increased sample sizes to be detected, (2) differences in the principal gradient between studies, (3) other incidental differences between our study and that of Hong et al. (2019) including differences in age, gender, resting state procedure, and cross-cultural differences, and (4) the choice of the AQ to measure autistic-like traits. These second, third and fourth points are considered in more detail below.

With regards to differences in the extracted principal gradient, in Hong et al. (2019) the principal gradient distribution consists of a higher peak resulting primarily from the combination of the visual, sensorimotor, and both attention networks, and a second less pronounced peak attributable to the control and default networks. By contrast, in our study we find that the sensorimotor and attention networks lie intermediate between the visual network and default/control networks. Note that an analysis of the HCP dataset using our decomposition parameters produced gradients more similar to those in Hong et al. (2019, see Supplementary Fig. 1). One contributing factor to the divergence in the decomposition results could be the difference between eyes-closed and eyes-open resting-state connectivity data. Our sample was instructed to close their eyes whilst the HCP data and part of the ABIDE data was collected during fixation. Closing the eyes has been linked to significant increases in connectivity of auditory and somatomotor networks to other networks (Agcaoglu et al., 2019) as well as decreased reliability (Patriat et al., 2013). It is possible that such differences additionally interact with autism status (Barttfeld et al., 2012).

The sample characteristics also differ between our study and that of Hong et al. (2019). Our sample was predominantly female (73%), over the age of 18, and was recruited in a Chinese university. Conversely, Hong et al. (2019) restricted their analysis to male participants from the ABIDE dataset (predominantly Western), and also included children. The functional connectome changes throughout development from late childhood to early adulthood (e.g., Oldham & Fornito, 2019). Within the gradient framework, there is a topographic reorganization of the connectome, transitioning from a principal gradient spanning from visual to somatomotor cortex in children to the principal gradient spanning from visual to transmodal cortex characteristic of adults, as unimodal regions reach their peak maturation first and transmodal regions last (Dong et al., 2021). This differentiation of the higher-order association cortices manifests as increases in eccentricity and overall gradient expansion (Park et al., 2021). However, it seems unlikely that this would have affected our findings of increased expansion related to sensory sensitivity, given the age homogeneity in our sample and the results of the multiple regression including age as a covariate (see Supplementary Materials). It is also unlikely to have contributed to our null results regarding the AQ, as Hong et al. (2019) report that, whilst disruptions in early systems occur in both children and adults, gradient reductions in the DMN are more pronounced in adults (Cohen’s d in children = 0.29, Cohen’s d in adults = 0.88). The fact that our sample consisted exclusively of young adults should have thus improved our chances of detecting any differences in gradient expansion, were these to extend to individual differences in AQ in the neurotypical population. The ASD literature as a whole suffers from gender bias, with females representing 10% of the mean sample size per resting state fMRI study evaluated in a recent review (Hull et al., 2017). However, fitting a multiple regression model with the distance between the default and visual network as the dependent variable and age, gender, AQ and GSQ scores as independent variables did not change the results (see Supplementary Materials). Likewise, the scarce literature on cross-cultural differences in the AQ suggests the culture of origin may need to be taken into consideration (Freeth et al., 2013; Lau et al., 2013). As in Lau et al. (2013), we find a negative correlation between the attention-to-detail subscale of the AQ and other subscales related to socio-communicative difficulties, which is generally positive in Western samples. Indeed, there is evidence of cross-cultural effects on attention allocation in the general population, whereby East Asians are more likely to attend holistically to the field, including background or contextual information, whilst Westerners analytically prioritise the focal, leading to corresponding behavioural dis/advantages in change blindness paradigms (Boduroglu et al., 2009; Masuda & Nisbett, 2006). Here, this variability has been attributed to both social structure (collectivistic vs individualistic) and a circular loop of more complex environments, aesthetic preference, and perception. In this context, it is plausible that higher attention-to-detail (a more Western attention style) is detrimental to social abilities in an East Asian environment, highlighting the importance of the interaction of individual traits and situational factors (Belmonte, 2020). In addition, it is conceivable that the association between sensory sensitivity and other aspects of the autism phenotype is weaker in certain cultures, as suggested by the smaller correlations found between the AQ and the GSQ in Japanese and Chinese samples (Ujiie & Wakabayashi, 2015; Ward et al., 2021), compared to the correlations found in Western samples (Horder et al., 2014; Robertson & Simmons, 2013).

As this is a non-clinical sample, the scales used to quantify autistic(-like) traits also differ between the studies. The AQ is the most extensively used scale in non-clinical autism research, which has resulted in a large body of literature using this measure. Although not developed as a diagnostic tool, it has been used to reliably discriminate between individuals with a diagnosis of ASD and typically developing individuals (Baron-Cohen et al., 2001). The AQ’s psychometric properties are however suboptimal. The factor structure, discriminative validity and Cronbach’s α (ranging from 0.54 to 0.88 in the Chinese version of the AQ used here) could be improved (Lau et al., 2013), and negatively phrased items show differential functioning between autistic and non-autistic samples (Agelink van Rentergem et al., 2019). Alternative measures of autistic(-like) traits applied often in research contexts include the Social Responsiveness Scale, which focuses on social communication (SRS-2 Constantino et al., 2003), to a lesser degree the Broader Autism Phenotype Questionnaire, designed to target the broader autistic phenotype (BAPQ; Hurley et al., 2007), and the recently developed Comprehensive Autistic Trait Inventory, which, as opposed to the previous scales, includes items addressing sensory sensitivity and physical repetitive behaviours (CATI; English et al., 2021). The choice of scales in this study (the AQ and GSQ in combination) has the advantage of measuring broader autistic-like traits and sensory sensitivities separately, which may have contributed to the detection of the specific association between the connectivity metrics and the GSQ.

Curiously, despite the discrepancies when comparing our gradient analysis results with those in Hong et al. (2019), our results would dovetail conceptually with the stepwise functional connectivity analysis in the same study, where they find an increased number of steps seeding from primary sensory cortices to default mode network in ASD. Cortical gradients have been described as a sequence of steps in connectivity space (Hong et al., 2019), and yet the two techniques do not necessarily yield identical results, as the methodologies themselves are not identical. For example, cortical gradient analysis features a dimensionality reduction step whilst stepwise functional connectivity does not, and stepwise functional connectivity accounts for indirect paths whilst cortical gradient analysis does not. Further studies assessing the effect of these inherent differences in the methodology would aid in the interpretation of the metrics derived from them.

On the other hand, gradient compression has been interpreted as a pattern that would render sensory input harder to ignore by not segregating it from internal processes, and thus compromising ‘higher-order’ cognitive processes (Hong et al., 2019; Roy & Uddin, 2021). It is worth noting that the changes in functional connectome hierarchy we report here are linked exclusively to sensory issues, and not to other autistic-like traits, such as social and communication deficits. It is therefore possible that we are comparing different cognitive profiles—one in which sensory issues tend to be more debilitating and are accompanied by the social and communication deficits characteristic of clinical autism, where the connectome hierarchy is compressed, and another non-clinical profile in which these experiences do not compromise higher-order functions, where the hierarchy is expanded. The question of whether autism should be framed as a spectrum disorder or a clearly demarcated diagnostic category remains a subject of debate (for a recent discussion of the pitfalls of an overly inclusive definition of autism ‘proper’, see Mottron & Bzdok, 2020). It will be important for future research to clarify if our null results with respect to the AQ hold or, alternatively, if there is a categorical difference between autistic-like traits and clinical autism with respect to functional connectivity gradients. Similarly, it will be important to confirm if our findings regarding sensory issues in a general population sample extend to clinical ASD.

In summary, this study shows the potential of the application of gradient decomposition methods to uncover individual differences in the general population as well as in clinical samples. We do not find evidence of an atypical connectome hierarchy using a broader definition of the autistic-like phenotype as measured by the AQ, suggesting that this instrument may not capture functional connectivity variability in the general population. Conversely, the expansion of the connectome hierarchy and particularly the segregation of the default and visual network connectivity patterns are linked to sensory sensitivity. Whilst caution is warranted in that the same behaviour can have different underpinnings (Chown & Leatherland, 2021; Happé & Frith, 2021), by the same token, this seems to be a strong argument for research using comprehensive transdiagnostic deep data (Lombardo et al., 2019). Future large-scale and deep studies in clinical and transdiagnostic contexts will be required to parse the multidimensional variability related to the heterogeneity of autistic and autistic-like traits.

### Supplementary Information

Below is the link to the electronic supplementary material.

Supplementary file1 (DOCX 256 kb)

## References

[CR1] Agcaoglu O, Wilson TW, Wang Y-P, Stephen J, Calhoun VD (2019). Resting state connectivity differences in eyes open versus eyes closed conditions. Human Brain Mapping.

[CR2] Agelink van Rentergem JA, Lever AG, Geurts HM (2019). Negatively phrased items of the Autism Spectrum Quotient function differently for groups with and without autism. Autism.

[CR3] Austin EJ (2005). Personality correlates of the broader autism phenotype as assessed by the Autism Spectrum Quotient (AQ). Personality and Individual Differences.

[CR4] Bar M, Aminoff E, Mason M, Fenske M (2007). The units of thought. Hippocampus.

[CR5] Baron-Cohen S, Leslie AM, Frith U (1985). Does the autistic child have a “theory of mind” ?. Cognition.

[CR6] Baron-Cohen S, Wheelwright S, Skinner R, Martin J, Clubley E (2001). The Autism-Spectrum Quotient (AQ): Evidence from Asperger syndrome/high-functioning autism, Malesand females, scientists and mathematicians. Journal of Autism and Developmental Disorders.

[CR7] Barttfeld P, Wicker B, Cukier S, Navarta S, Lew S, Leiguarda R, Sigman M (2012). State-dependent changes of connectivity patterns and functional brain network topology in autism spectrum disorder. Neuropsychologia.

[CR8] Belluscio BA, Jin L, Watters V, Lee TH, Hallett M (2011). Sensory sensitivity to external stimuli in Tourette syndrome patients. Movement Disorders.

[CR9] Belmonte MK (2020). Other and other waters in the river: Autism and the futility of prediction. Behavioral and Brain Sciences.

[CR10] Benedek M, Jauk E, Beaty RE, Fink A, Koschutnig K, Neubauer AC (2016). Brain mechanisms associated with internally directed attention and self-generated thought. Scientific Reports.

[CR11] Bertelsen N, Landi I, Bethlehem RAI, Seidlitz J, Busuoli EM, Mandelli V, Satta E, Trakoshis S, Auyeung B, Kundu P, Loth E, Dumas G, Baumeister S, Beckmann CF, Bölte S, Bourgeron T, Charman T, Durston S, Ecker C, Lombardo MV (2021). Imbalanced social-communicative and restricted repetitive behavior subtypes of autism spectrum disorder exhibit different neural circuitry. Communications Biology.

[CR12] Bethlehem, R. A. I., Paquola, C., Seidlitz, J., Ronan, L., Bernhardt, B., Consortium, C.-C., & Tsvetanov, K. A. (2020). Dispersion of functional gradients across the lifespan. *BioRxiv*, 2020.02.27.968537. 10.1101/2020.02.27.968537 10.1016/j.neuroimage.2020.117299 PMC7779368 32828920

[CR13] Bijlenga D, Tjon-Ka-Jie JYM, Schuijers F, Kooij JJS (2017). Atypical sensory profiles as core features of adult ADHD, irrespective of autistic symptoms. European Psychiatry.

[CR14] Binder JR, Desai RH, Graves WW, Conant LL (2009). Where is the semantic system? A critical review and meta-analysis of 120 functional neuroimaging studies. Cerebral Cortex.

[CR15] Boduroglu A, Shah P, Nisbett RE (2009). Cultural differences in allocation of attention in visual information processing. Journal of Cross-Cultural Psychology.

[CR16] Brock J (2012). Alternative Bayesian accounts of autistic perception: Comment on Pellicano and Burr. Trends in Cognitive Sciences.

[CR86] Brown C, Tollefson N, Dunn W, Cromwell R, Filion D (2001). The adult sensory profile: Measuring patterns of sensory processing. The American Journal of Occupational Therapy.

[CR17] Chown N, Leatherland J (2021). Can a person be ‘A Bit Autistic’? A response to Francesca Happé and Uta Frith. Journal of Autism and Developmental Disorders.

[CR18] Christoff K, Gordon AM, Smallwood J, Smith R, Schooler JW (2009). Experience sampling during fMRI reveals default network and executive system contributions to mind wandering. Proceedings of the National Academy of Sciences.

[CR19] Constantino JN, Davis SA, Todd RD, Schindler MK, Gross MM, Brophy SL, Metzger LM, Shoushtari CS, Splinter R, Reich W (2003). Validation of a Brief Quantitative Measure of Autistic Traits: Comparison of the social responsiveness scale with the autism diagnostic interview-revised. Journal of Autism and Developmental Disorders.

[CR20] Constantino JN, Todd RD (2003). Autistic traits in the general population: A twin study. Archives of General Psychiatry.

[CR21] Di Martino A, Ross K, Uddin LQ, Sklar AB, Castellanos FX, Milham MP (2009). Functional brain correlates of social and nonsocial processes in autism spectrum disorders: An activation likelihood estimation meta-analysis. Biological Psychiatry.

[CR22] Dong H-M, Margulies DS, Zuo X-N, Holmes AJ (2021). Shifting gradients of macroscale cortical organization mark the transition from childhood to adolescence. Proceedings of the National Academy of Sciences.

[CR23] English MCW, Gignac GE, Visser TAW, Whitehouse AJO, Enns JT, Maybery MT (2021). The Comprehensive Autistic Trait Inventory (CATI): Development and validation of a new measure of autistic traits in the general population. Molecular Autism.

[CR24] Fischl B (2012). FreeSurfer. NeuroImage.

[CR25] Freeth M, Sheppard E, Ramachandran R, Milne E (2013). A cross-cultural comparison of autistic traits in the UK, India and Malaysia. Journal of Autism and Developmental Disorders.

[CR26] Green SA, Hernandez L, Bookheimer SY, Dapretto M (2016). Salience network connectivity in autism is related to brain and behavioral markers of sensory overresponsivity. Journal of the American Academy of Child & Adolescent Psychiatry.

[CR27] Gusnard DA, Akbudak E, Shulman GL, Raichle ME (2001). Medial prefrontal cortex and self-referential mental activity: Relation to a default mode of brain function. Proceedings of the National Academy of Sciences.

[CR28] Happé F, Frith U (2006). The weak coherence account: Detail-focused cognitive style in autism spectrum disorders. Journal of Autism and Developmental Disorders.

[CR29] Happé F, Frith U (2021). Dimensional or categorical approaches to autism? Both are needed. A reply to Nick Chown and Julia Leatherland. Journal of Autism and Developmental Disorders.

[CR30] He, J., Williams, Z., Harris, A. D., Powell, H., Schaaf, R., Tavassoli, T., & Puts, N. (2022). *A working taxonomy for describing the sensory differences of autism*. PsyArXiv. 10.31234/osf.io/jmv6k 10.1186/s13229-022-00534-1 PMC10091684 37041612

[CR31] Hong S-J, Vos de Wael R, Bethlehem RAI, Lariviere S, Paquola C, Valk SL, Milham MP, Di Martino A, Margulies DS, Smallwood J, Bernhardt BC (2019). Atypical functional connectome hierarchy in autism. Nature Communications.

[CR32] Hong S-J, Xu T, Nikolaidis A, Smallwood J, Margulies DS, Bernhardt B, Vogelstein J, Milham MP (2020). Toward a connectivity gradient-based framework for reproducible biomarker discovery. NeuroImage.

[CR33] Horder J, Wilson CE, Mendez MA, Murphy DG (2014). Autistic traits and abnormal sensory experiences in adults. Journal of Autism and Developmental Disorders.

[CR34] Hull JV, Dokovna LB, Jacokes ZJ, Torgerson CM, Irimia A, Van Horn JD (2017). Resting-state functional connectivity in autism spectrum disorders: A review. Frontiers in Psychiatry.

[CR35] Hurley RSE, Losh M, Parlier M, Reznick JS, Piven J (2007). The Broad Autism Phenotype Questionnaire. Journal of Autism and Developmental Disorders.

[CR36] Iacoboni M, Lieberman MD, Knowlton BJ, Molnar-Szakacs I, Moritz M, Throop CJ, Fiske AP (2004). Watching social interactions produces dorsomedial prefrontal and medial parietal BOLD fMRI signal increases compared to a resting baseline. NeuroImage.

[CR37] JaoKeehn RJ, Pueschel EB, Gao Y, Jahedi A, Alemu K, Carper R, Fishman I, Müller R-A (2021). Underconnectivity between visual and salience networks and links with sensory abnormalities in autism spectrum disorders. Journal of the American Academy of Child & Adolescent Psychiatry.

[CR38] Jenkinson M, Bannister P, Brady M, Smith S (2002). Improved optimization for the robust and accurate linear registration and motion correction of brain images. NeuroImage.

[CR39] Jenkinson, M., Pechaud, M., & Smith, S. (2005). *BET2—MR-Based Estimation of Brain, Skull and Scalp Surfaces*. 1.

[CR40] Jenkinson M, Smith S (2001). A global optimisation method for robust affine registration of brain images. Medical Image Analysis.

[CR42] Lau WY-P, Gau SS-F, Chiu Y-N, Wu Y-Y, Chou W-J, Liu S-K, Chou M-C (2013). Psychometric properties of the Chinese version of the Autism Spectrum Quotient (AQ). Research in Developmental Disabilities.

[CR43] Little LM, Dean E, Tomchek SD, Dunn W (2017). Classifying sensory profiles of children in the general population. Child: Care, Health and Development.

[CR44] Liu W, Wei D, Chen Q, Yang W, Meng J, Wu G, Bi T, Zhang Q, Zuo X-N, Qiu J (2017). Longitudinal test-retest neuroimaging data from healthy young adults in southwest China. Scientific Data.

[CR45] Lombardo MV, Lai M-C, Baron-Cohen S (2019). Big data approaches to decomposing heterogeneity across the autism spectrum. Molecular Psychiatry.

[CR46] Margulies DS, Ghosh SS, Goulas A, Falkiewicz M, Huntenburg JM, Langs G, Bezgin G, Eickhoff SB, Castellanos FX, Petrides M, Jefferies E, Smallwood J (2016). Situating the default-mode network along a principal gradient of macroscale cortical organization. Proceedings of the National Academy of Sciences.

[CR47] Masuda T, Nisbett RE (2006). Culture and change blindness. Cognitive Science.

[CR48] Mckeown B, Strawson WH, Wang H-T, Karapanagiotidis T, Vos de Wael R, Benkarim O, Turnbull A, Margulies D, Jefferies E, McCall C, Bernhardt B, Smallwood J (2020). The relationship between individual variation in macroscale functional gradients and distinct aspects of ongoing thought. NeuroImage.

[CR49] Mesulam M (2012). The evolving landscape of human cortical connectivity: Facts and inferences. NeuroImage.

[CR50] Mottron L, Bzdok D (2020). Autism spectrum heterogeneity: Fact or artifact?. Molecular Psychiatry.

[CR51] Mottron L, Dawson M, Soulières I, Hubert B, Burack J (2006). Enhanced perceptual functioning in autism: An update, and eight principles of autistic perception. Journal of Autism and Developmental Disorders.

[CR52] Murphy C, Jefferies E, Rueschemeyer S-A, Sormaz M, Wang H, Margulies DS, Smallwood J (2018). Distant from input: Evidence of regions within the default mode network supporting perceptually-decoupled and conceptually-guided cognition. NeuroImage.

[CR53] Oldham S, Fornito A (2019). The development of brain network hubs. Developmental Cognitive Neuroscience.

[CR54] Palmer CJ, Lawson RP, Hohwy J (2017). Bayesian approaches to autism: Towards volatility, action, and behavior. Psychological Bulletin.

[CR55] Park, B., Bethlehem, R. A., Paquola, C., Larivière, S., Rodríguez-Cruces, R., Vos de Wael, R., Neuroscience in Psychiatry Network (NSPN) Consortium, Bullmore, E. T., & Bernhardt, B. C (2021). An expanding manifold in transmodal regions characterizes adolescent reconfiguration of structural connectome organization. eLife.

[CR56] Patriat R, Molloy EK, Meier TB, Kirk GR, Nair VA, Meyerand ME, Prabhakaran V, Birn RM (2013). The effect of resting condition on resting-state fMRI reliability and consistency: A comparison between resting with eyes open, closed, and fixated. NeuroImage.

[CR58] Pellicano E, Burr D (2012). When the world becomes ‘too real’: A Bayesian explanation of autistic perception. Trends in Cognitive Sciences.

[CR59] Raichle ME, MacLeod AM, Snyder AZ, Powers WJ, Gusnard DA, Shulman GL (2001). A default mode of brain function. Proceedings of the National Academy of Sciences.

[CR60] Robertson AE, Simmons DR (2013). The relationship between sensory sensitivity and autistic traits in the general population. Journal of Autism and Developmental Disorders.

[CR61] Robertson AE, Simmons DR (2015). The sensory experiences of adults with autism spectrum disorder: A qualitative analysis. Perception.

[CR62] Roy D, Uddin LQ (2021). Atypical core-periphery brain dynamics in autism. Network Neuroscience.

[CR63] Rugg MD, Vilberg KL (2013). Brain networks underlying episodic memory retrieval. Current Opinion in Neurobiology.

[CR64] Ruzich E, Allison C, Smith P, Watson P, Auyeung B, Ring H, Baron-Cohen S (2015). Measuring autistic traits in the general population: A systematic review of the Autism-Spectrum Quotient (AQ) in a nonclinical population sample of 6,900 typical adult males and females. Molecular Autism.

[CR65] Sapey-Triomphe L-A, Moulin A, Sonié S, Schmitz C (2018). The Glasgow Sensory Questionnaire: Validation of a French language version and refinement of sensory profiles of people with high autism-spectrum quotient. Journal of Autism and Developmental Disorders.

[CR66] Schaefer A, Kong R, Gordon EM, Laumann TO, Zuo X-N, Holmes AJ, Eickhoff SB, Yeo BTT (2018). Local-global parcellation of the human cerebral cortex from intrinsic functional connectivity MRI. Cerebral Cortex.

[CR68] Schulz SE, Stevenson RA (2022). Convergent validity of behavioural and subjective sensitivity in relation to autistic traits. Journal of Autism and Developmental Disorders.

[CR69] Sinha P, Kjelgaard MM, Gandhi TK, Tsourides K, Cardinaux AL, Pantazis D, Diamond SP, Held RM (2014). Autism as a disorder of prediction. Proceedings of the National Academy of Sciences.

[CR70] Smallwood J, Bernhardt BC, Leech R, Bzdok D, Jefferies E, Margulies DS (2021). The default mode network in cognition: A topographical perspective. Nature Reviews Neuroscience.

[CR71] Smith SM (2002). Fast robust automated brain extraction. Human Brain Mapping.

[CR72] Smith SM, Jenkinson M, Woolrich MW, Beckmann CF, Behrens TEJ, Johansen-Berg H, Bannister PR, De Luca M, Drobnjak I, Flitney DE, Niazy RK, Saunders J, Vickers J, Zhang Y, De Stefano N, Brady JM, Matthews PM (2004). Advances in functional and structural MR image analysis and implementation as FSL. NeuroImage.

[CR73] Spreng RN, Gerlach KD, Turner GR, Schacter DL (2015). Autobiographical planning and the brain: Activation and its modulation by qualitative features. Journal of Cognitive Neuroscience.

[CR74] Ujiie Y, Wakabayashi A (2015). Psychometric properties and overlap of the GSQ and AQ among Japanese University Students. International Journal of Psychological Studies.

[CR75] Valk, S. L., Kanske, P., Park, B., Hong, S. J., Böckler-Raettig, A., Trautwein, F.-M., Bernhardt, B. C., & Singer, T. (2021). Changing the social brain: Plasticity along macro-scale axes of functional connectivity following social mental training. *BioRxiv*, 2020.11.11.377895. 10.1101/2020.11.11.377895

[CR76] Van de Cruys S, de-Wit L, Evers K, Boets B, Wagemans J (2013). Weak priors versus overfitting of predictions in autism: Reply to Pellicano and Burr (TICS, 2012). I-Perception.

[CR77] Van Hulle CA, Schmidt NL, Goldsmith HH (2012). Is sensory over-responsivity distinguishable from childhood behavior problems? A phenotypic and genetic analysis. Journal of Child Psychology and Psychiatry, and Allied Disciplines.

[CR78] Verhoeff B (2013). Autism in flux: A history of the concept from Leo Kanner to DSM-5. History of Psychiatry.

[CR79] Vos de Wael R, Benkarim O, Paquola C, Lariviere S, Royer J, Tavakol S, Xu T, Hong S-J, Langs G, Valk S, Misic B, Milham M, Margulies D, Smallwood J, Bernhardt BC (2020). BrainSpace: A toolbox for the analysis of macroscale gradients in neuroimaging and connectomics datasets. Communications Biology.

[CR80] de Wael RV, Larivière S, Caldairou B, Hong S-J, Margulies DS, Jefferies E, Bernasconi A, Smallwood J, Bernasconi N, Bernhardt BC (2018). Anatomical and microstructural determinants of hippocampal subfield functional connectome embedding. Proceedings of the National Academy of Sciences.

[CR81] Ward J (2019). Individual differences in sensory sensitivity: A synthesizing framework and evidence from normal variation and developmental conditions. Cognitive Neuroscience.

[CR82] Ward J, Brown P, Sherwood J, Simner J (2017). An autistic-like profile of attention and perception in synaesthesia. Cortex.

[CR83] Ward J, Hoadley C, Hughes JEA, Smith P, Allison C, Baron-Cohen S, Simner J (2017). Atypical sensory sensitivity as a shared feature between synaesthesia and autism. Scientific Reports.

[CR84] Ward J, Ren Z, Qiu J (2021). Autistic traits in the neurotypical Chinese population: A Chinese version of Glasgow sensory questionnaire and a cross-cultural difference in attention-to-detail. Journal of Autism and Developmental Disorders.

[CR85] Xia, M., Liu, J., Sun, X., Ma, Q., Wang, X., Wei, D., Chen, Y., Liu, B., Huang, C.-C., Zheng, Y., Wu, Y., Chen, T., Cheng, Y., Xu, X., Gong, Q., Si, T., Qiu, S., Lin, C.-P., Cheng, J., … Group, D.-M. D. D. W. (2020). Large-scale gradient dysfunction of the functional connectome in major depression. *BioRxiv*, 2020.10.24.352153. 10.1101/2020.10.24.352153

