# Supplementary material for: Higher Sensory Sensitivity is Linked to Greater Expansion Amongst Functional Connectivity Gradients
Source: J Autism Dev Disord. 2022 Oct 13;54(1):56–74. doi: 10.1007/s10803-022-05772-z (PMC10791721; doi:10.1007/s10803-022-05772-z)
Supplement: Supplementary file 1 — Supplementary file1 (DOCX 256 kb) [file 10803_2022_5772_MOESM1_ESM.docx]

Supplementary Materials

*Correspondence across samples*

The correspondence between our sample’s individual-level and group-level first three gradients (see Table 1) is substantially higher than the correspondence between our sample’s individual-level gradients and the HCP-derived gradients and between our sample’s group-level gradients and the HCP-derived gradients for the first three gradients (Tables 2 and 3, respectively). This is to be expected given the lack of alignment to a common template.

In terms of the gradient cortical topography, we partially replicate the results found in the literature (e.g., Hong et al., 2019; Margulies et al., 2016). (See Supplementary Figure 1 for a comparison with the gradients derived from HCP data freely available in the Brainspace toolbox; Vos de Wael et al., 2020). In the present sample, the somatomotor cortex is closer to the centre of the gradient whereas others have reported it as closer to the extreme end of the gradient alongside visual cortex, which can be attributed to, e.g., differences in the connectome resolution and decomposition methods (diffusion embedding vs PCA).

*Multiple regression model comparison – effect of age and gender:*

A multiple regression model indicates that AQ and GSQ scores significantly predict the distance along the principal gradient between visual and default networks (F(2, 367) = 4.986, p = 0.007, adjusted R^2^ = .0212), with only GSQ adding significantly to the prediction (p = 0.003). An ANOVA comparing this model to a second model including age and gender as independent variables indicated that this did not improve the prediction (F(2, 365)= 1.578, p = 0.208). Furthermore, bootstrapped CIs based on 1000 replications calculated using the boot package in R (Canty & Ripley, 2021) include 0 for all regressors except GSQ (age: [-0.150, 0.042], gender: [-0.044, 0.416], AQ: [-0.067, 0.131], GSQ: [0.051, 0.272]).

*Multivariate analysis*

The association between individual differences in autistic traits and connectivity patterns was tested using a multivariate regression model following the approach in Mckeown et al. (2020). We calculated Spearman’s rank correlation between each of the first three group-level gradients and each of the first three individual-level gradients, resulting in three similarity scores per participant. These scores were z-transformed and then entered as dependent variables in a multivariate regression analysis with AQ subscale scores and GSQ scores as independent variables, and age and gender as covariates (see Supplementary Table 4 for the correlation matrix of total GSQ scores and AQ subscale scores). Neither AQ or GSQ is a significant predictor of similarity scores. However, decomposing the AQ into its five subscales revealed a multivariate effect of the attention-to-detail subscale (Type II MANOVA: Pillai’s trace = 0.028, F(3,359) = 3.508, p = 0.016). To follow up on this effect, we extracted the corresponding parameter estimates – the only association where the confidence interval does not include zero is with the third gradient (first gradient (β = 0.002, 95% CI = [-0.006, 0.010], p = 0.654), second gradient (β = -0.004, 95% CI = [-0.012 0.003], p = 0.351), third gradient (β = -0.010, 95% CI = [-0.017 -0.003], p = 0.023).

The multivariate similarity analysis indicates that the individual differences related to sensory sensitivity are not reflected in individual differences in functional motifs. Conversely, these do correlate with variability in the attention-to-detail subscale along the third gradient. Given the topography of this gradient, which spans from the ventral attention to the somatomotor network, this is not entirely surprising. Please note that other studies using gradient decomposition methods find a third gradient which reflects the segregation of default-mode and task-positive regions (Mckeown et al., 2020), yet in our sample this pattern is found in the fourth gradient, such that these two components seem reversed in our PCA analysis.


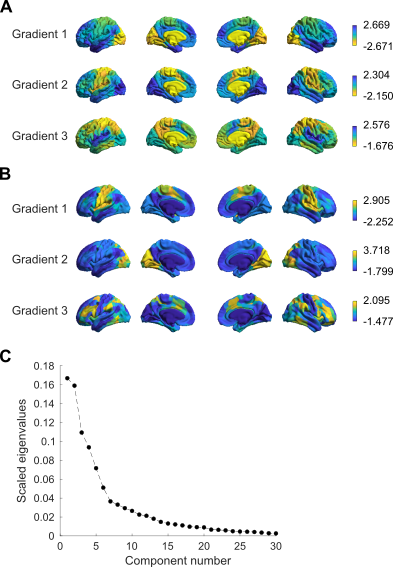


*Supplementary Figure 1.* **A** First three gradients derived from our sample’s group-average functional connectivity matrix, using PCA as the dimension reduction technique, a sparsity parameter of 0.95 and a cosine similarity kernel, **B** the HCP-derived first three gradients, extracted using the same parameters, for comparison (bottom right). Regions with similar connectivity patterns are in close proximity along the gradient and thus share similar colours. **C** Scree plot of the scaled eigenvalues (explained variance) after the decomposition of our sample’s group-average functional connectivity matrix


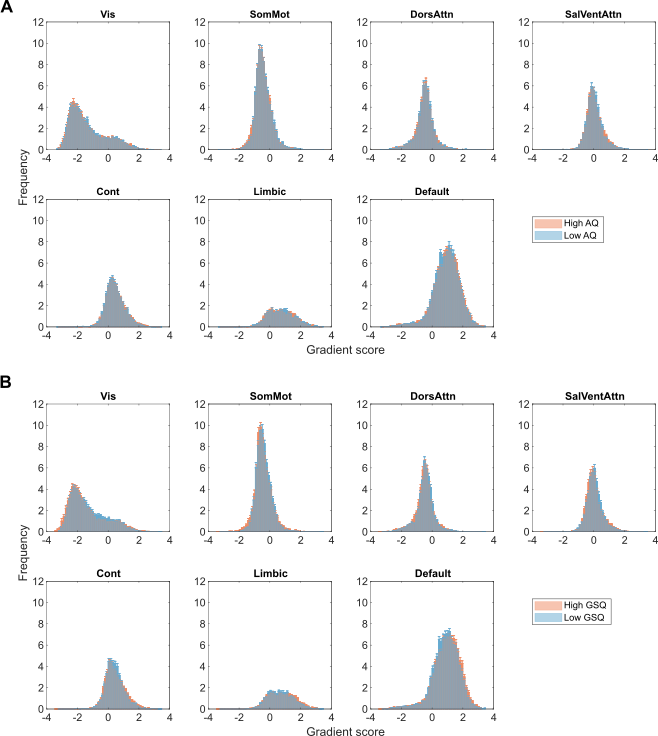


*Supplementary Figure 2.* Histogram of the principal gradient scores, segregated into the 7 networks identified by the Schaefer atlas, for **A** participants with high AQ (AQ>23, n=94), and participants with low AQ (AQ<17, n=118), and **B** participants with high GSQ (GSQ>58, n=122), and participants with low GSQ (GSQ<42, n=121) as per a tercile split. Regions with similar connectivity patterns are in close proximity along the gradient and thus have similar gradient scores (Vis = visual, SomMot = somatomotor, DorsAttn = dorsal attention, SalVentAttn = salience-ventral attention, Cont = control, Limbic = limbic, Default = default mode network). Error bars represent the standard error of the mean bin count

Supplementary Table 1: Absolute minimum, absolute maximum, mean and SD of the z-transformed Spearman rank correlation between the first three group-average gradients and each of the first three individual-level aligned gradients derived from our sample.

|  | Gradient 1 | Gradient 2 | Gradient 3 |
| --- | --- | --- | --- |
| Absolute minimum ρ_s_ | 0.614 | 0.717 | 0.484 |
| Absolute maximum ρ_s_ | 1.704 | 1.771 | 1.454 |
| Mean ρ_s_ | 1.277 | 1.315 | 1.030 |
| SD ρ_s_ | 0.186 | 0.175 | 0.165 |

Supplementary Table 2: Absolute minimum, absolute maximum, mean and SD of the z-transformed Spearman rank correlation between the first three group-average gradients derived from the HCP data and each of the first three individual-level aligned gradients derived from our sample.

|  | Gradient 1 | Gradient 2 | Gradient 3 |
| --- | --- | --- | --- |
| Absolute minimum ρ_s_ | 0.052 | 0.111 | 0.006 |
| Absolute maximum ρ_s_ | 0.357 | 0.417 | 0.271 |
| Mean ρ_s_ | 0.222 | 0.311 | 0.140 |
| SD ρ_s_ | 0.049 | 0.048 | 0.048 |

Supplementary Table 3: z-transformed Spearman rank correlation between group-average gradients 1-3 derived from the HCP data and the group-average aligned gradients 1-3

|  | Gradient 1 | Gradient 2 | Gradient 3 |
| --- | --- | --- | --- |
| ρ_s_ | 0.226 | 0.377 | 0.18089 |

Supplementary Table 4: Spearman’s rank correlation matrix for AQ subscale scores and total GSQ scores. * p <0.05, ** p <0.01, *** p <0.001

|  | Social | Attention switching | Communication | Imagination | GSQ |
| --- | --- | --- | --- | --- | --- |
| Attention to detail | **-0.234***** | **-0.140**** | **-0.202***** | -0.093 | **0.124*** |
| Social |  | **0.337***** | **0.473***** | **0.324***** | 0.098 |
| Attention switching |  |  | **0.307***** | 0.078 | 0.064 |
| Communication |  |  |  | **0.315***** | **0.243***** |
| Imagination |  |  |  |  | 0.092 |
